# Efficacy and Safety of the Surgery-First Approach Compared to the Chemotherapy-First Approach for Treating Low-Risk Gestational Trophoblastic Neoplasia: A Systematic Review and Meta-Analysis

**DOI:** 10.7759/cureus.45726

**Published:** 2023-09-21

**Authors:** Kyosuke Kamijo, Kentaro Ishida, Shiho Oide, Keisuke Anan, Shunsuke Taito, Yuki Kataoka, Kenro Chikazawa

**Affiliations:** 1 Obstetrics and Gynecology, Nagano Manicipal Hospital, Nagano, JPN; 2 Department of Obstetrics and Gynecology, Nagano Prefectural Kiso Hospital, Kiso-gun, JPN; 3 Department of Systematic Reviewers, Scientific Research Works Peer Support Group (SRWS-PSG), Osaka, JPN; 4 Department of Obstetrics and Gynaecology, Osaka Red Cross Hospital, Osaka, JPN; 5 Department of Systematic Reviewers, Scientific Research WorkS Peer Support Group (SRWS-PSG), Osaka, JPN; 6 Urogynecology Center, Kameda Medical Center, Chiba, JPN; 7 Division of Respiratory Medicine, Saiseikai Kumamoto Hospital, Kumamoto, JPN; 8 Department of Healthcare Epidemiology, Kyoto University Graduate School of Medicine/School of Public Health, Kyoto, JPN; 9 Division of Rehabilitation, Hiroshima University Hospital, Hiroshima, JPN; 10 Department of Internal Medicine, Kyoto Min-iren Asukai Hospital, Kyoto, JPN; 11 Department of Community Medicine, Kyoto University Graduate School of Medicine/Section of Clinical Epidemiology, Kyoto, JPN; 12 Department of Obstetrics and Gynecology, Jichi Medical University, Saitama Medical Center, Saitama, JPN

**Keywords:** trophoblastic neoplasms, hysterectomy, gestational trophoblastic disease, contraception, chemotherapy

## Abstract

For gestational trophoblastic neoplasia (GTN) affecting women of reproductive age, the chemotherapy-first approach is often preferred over the surgery-first approach. Low-risk GTN is treated with a chemotherapy-first approach, but the number of courses required can affect fertility. A surgery-first approach may decrease the number of chemotherapy courses, but its efficacy and safety compared to a chemotherapy-first approach are unclear. Thus, we investigated the efficacy and safety of the surgery-first approach compared to the chemotherapy-first approach in treating low-risk GTN.

We searched the MEDLINE, Embase, Cochrane Central Register of Controlled Trials, ClinicalTrials.gov, and World Health Organization International Clinical Trials Registry Platform databases for relevant articles in July 2023. A systematic review and meta-analysis of outcome measures were conducted using a random-effects model. The primary outcomes were remission, the mean number of chemotherapy courses required to cure, and adverse events. The certainty of the evidence was evaluated using the Grading of Recommendations, Assessment, Development, and Evaluation approach. This study protocol was registered in the Open Science Framework (https://osf.io/kysvn/).

Studies for low-risk GTN included a qualitative synthesis (with 2,192 participants and ten studies, eight of which were about second uterine curettage and two about hysterectomy) and a meta-analysis (with 138 participants and two randomized controlled trials (RCTs) that compared first-line treatments of second uterine curettage and chemotherapy). Second uterine curettage may result in little to no difference in remission (risk ratio: 1.00, 95% confidence interval: 0.96-1.05; low certainty) and a slight reduction in adverse events (risk ratio: 0.87, 95% confidence interval: 0.47-1.60; low certainty). The evidence is very uncertain on the mean number of chemotherapy courses (mean difference: 2.84 lower, 95% confidence interval: 7.31 lower to 1.63 higher; very low certainty).

Based on clinical outcomes, second uterine curettage can be comparable to the chemotherapy-first approach as a first-line treatment option for low-risk GTN; however, the overall certainty of the evidence was low or very low.

## Introduction and background

Gestational trophoblastic neoplasia (GTN), which is not a rare disease, can affect women of reproductive age worldwide; despite its prevalence, optimal treatment options remain under investigation [1]. For patients with low-risk GTN diagnosed using risk scoring (<7) [2,3], chemotherapy-first approaches, such as administration of several single-agent chemotherapy courses along with multi-agent chemotherapy or surgical interventions, are the standard treatment and result in remission rates approaching 100% [4,5]. However, the number of chemotherapy cycles required to cure GTN is a major concern for women due to the impact on future fertility and overall drug toxicity [1,6-8].

The surgery-first approach is another treatment option to decrease the number of chemotherapy courses required [1,6,9]. Several randomized controlled trials (RCTs) and observational studies have assessed the efficacy and safety of surgery-first approaches, such as second uterine curettage or hysterectomy plus chemotherapy, compared with those of the chemotherapy-first approach for the treatment of low-risk GTN [10-16]. However, the efficacy and safety of the surgery-first approach have not been systematically reviewed. This systematic review and meta-analysis aimed to summarize the available evidence on the efficacy and safety of the surgery-first approach compared to the chemotherapy-first approach to treat low-risk GTN.

## Review

Materials and methods

This systematic review was conducted according to the Preferred Reporting Items for Systematic Reviews and Meta-Analyses (PRISMA) 2020 guidelines and assessed using the PRISMA 2020 checklist [17]. The protocol for this study was published in the Open Science Framework [18].

The surgery-first approach was defined as a second uterine curettage or hysterectomy plus chemotherapy as required. In contrast, the chemotherapy-first approach was defined as single-agent chemotherapy plus multi-agent chemotherapy or surgical interventions as required.

Study Selection

We included all published and unpublished articles on RCTs and observational studies in which interventions for the treatment of low-risk GTN were assessed. In addition, we included papers on crossover trials, quasi-experimental studies, quasi-randomized trials, conference abstracts, and letters. We did not set language or country restrictions or exclude studies based on the observation period or publication year. Non-comparative studies, including case series and case reports, were excluded. Patients with low-risk GTN, as defined by the International Federation of Gynaecology and Obstetrics (FIGO)/WHO staging and risk scoring system [2,3], who received second uterine curettage, hysterectomy, or a chemotherapy-first approach, were included. We excluded patients with high-risk GTN, placental site trophoblastic tumors, epithelial trophoblastic tumors, and those with evidence of metastasis or recurrent GTN that had previously been treated using chemotherapy. The interventions examined included second uterine curettage, hysterectomy, and the chemotherapy-first approach. The chemotherapy-first approach was defined as administering chemotherapeutic agents (e.g., methotrexate [MTX], actinomycin D, fluorouracil, etoposide, dactinomycin, or carboplatin) at any dose, duration, frequency, or setting as the first-line treatment for GTN. The primary outcomes assessed were remission, the mean number of chemotherapy courses required to cure, and adverse events. Remission was defined as the conversion of positive serum or urine hCG results to negative. The mean number of chemotherapy courses required to cure included consolidation courses administered after the normalization of hCG. This measure was counted as zero in cases of normalization of hCG after the second uterine curettage or hysterectomy. Adverse events were defined based on the definitions of the authors of the included studies; the adverse events included drug toxicity (fatigue, anemia, nausea, and alopecia) [19], uterine perforation, and unexpected surgical interventions, including emergency hysterectomy and uterine curettage.

On November 9, 2021, we searched the MEDLINE (via PubMed), Embase (via ProQuest), Cochrane Central Register of Controlled Trials, Clinicaltrials.gov, and World Health Organization (WHO) International Clinical Trials Registry Platform (ICTRP) databases for relevant articles. We updated on July 14, 2023, and also screened the reference lists of clinical guidelines and the articles included in the qualitative synthesis for additional relevant literature by hand searching. The full search strategy is presented in Appendix Table 3. Two of the three reviewers (KK, KI, and SO) independently screened the titles and abstracts of the articles to determine if they met the inclusion criteria. After screening the abstracts, the full texts of potentially eligible articles were retrieved, and each reviewer independently selected the eligible studies. We contacted the authors of the articles if relevant data were missing. Disagreements between the two reviewers were resolved through consultation with a third reviewer (KA).

Data Extraction

The authors of the papers were contacted to obtain more information if the studies lacked certain information. Two of the three independent reviewers (KK, KI, and SO) extracted data from the articles using a data collection form. The extracted data included the study design, population, interventions, outcome measurement, and results. Disagreements between the two reviewers were resolved through consultation with a third reviewer (KA).

Quality Assessment 

Different tools were used to address the two types of studies (randomized and non-randomized). Two of the three reviewers (KK, KI, and SO) independently evaluated the risk of bias in the studies using the risk of bias (RoB) 2 tool [20] and the risk of bias in non-randomized studies of interventions (ROBINS-I) [21]. ROBINS-I was utilized after setting the FIGO score, stage, pre-intervention hCG level, and the patient’s intention as the confounding domains, with no co-interventions. Disagreements regarding data extraction and quality assessment were settled through consultation with a third reviewer (KA).

Statistical Analysis

All analyses were conducted using RevMan 5.4 (The Nordic Cochrane Centre, Cochrane Collaboration, Copenhagen, Denmark). The meta-analysis was performed using a random-effects model. Relative risk (RR) and 95% confidence intervals (CIs) were calculated for bivariate variables (remission and adverse events), and the mean difference (MD) and 95% CIs were calculated for continuous variables (mean number of chemotherapy courses required to cure) using the methodology outlined in the Cochrane Handbook [22]. We estimated the missing mean and the standard deviation [23]. The results for each study were tabulated and visualized using forest plots.

For missing data and statistics, we performed a per-protocol analysis for all dichotomous and continuous data, as much as possible, after imputing the missing data [24]. Subsequently, we performed a meta-analysis of the data in the original studies. We excluded studies from the meta-analysis if they were assessed as at critical risk of bias based on ROBINS-I [21]. Regarding the assessment of heterogeneity, we evaluated statistical heterogeneity through visual inspection of the forest plots and calculated the I2 statistic, and when substantial heterogeneity (I2> 50%) was noted, we assessed its reason [22]. To assess reporting bias, we searched the clinical trial registry systems (ClinicalTrials.gov and ICTRP) and performed an extensive literature search for unpublished trials.

Summary of the Findings 

Two reviewers (KK and KA) independently summarized the findings for the primary outcomes according to the guidelines in the Cochrane Handbook [22]. The assessment of the findings included overall grading of the evidence quality, collated using the Grading of Recommendations Assessment, Development, and Evaluation (GRADE) approach [22,25]. Disagreements were resolved through consultation with a third reviewer (ST).

Differences Between the Study Protocol and the Review

Owing to insufficient data, the comparison of second uterine curettage with or without chemotherapy, hysterectomy with or without chemotherapy, and chemotherapy was not performed. We had planned to perform a network meta-analysis to consider three different approaches (chemotherapy, second uterine curettage, and hysterectomy), aiming to propose an approach in line with individual fertility preservation desires; however, owing to the lack of eligible studies in the hysterectomy with or without chemotherapy, we could not perform this. In the qualitative synthesis, we included studies that did not provide complete information on the risk-scoring system used for low-risk GTN. Only one study assessed remission and adverse events as primary outcomes after a second uterine curettage [11]. Hence, we did not perform a meta-analysis, heterogeneity assessment, and subgroup and sensitivity analyses of remission and adverse events as primary outcomes. 

Similarly, due to insufficient data, we did not conduct a subgroup analysis of the presence or absence of metastasis (stages I, II, III, or IV) [1], risk score (≤4 or ≥5) [1,26], and exclusion of additional treatment chemotherapy cycles past normalization of hCG [27-29] in evaluating the influence of the effect modifier, or sensitivity analysis for missing participants to verify the robustness of the results by seeking informative missingness odds ratios in the mean number of chemotherapy courses required for a cure [30]. We did not conduct a funnel plot analysis or an Egger’s test because we found fewer than 10 trials with similar sample sizes [22]. 

In the final meta-analysis, we exclusively evaluated the mean number of chemotherapy courses required to cure and conducted a subgroup analysis that excluded studies on second uterine curettage without chemotherapy, as well as three sensitivity analyses that respectively omitted studies with imputed statistics, non-standard chemotherapy protocols, and a high risk of bias.

Results

Search Results

**Figure 1:**
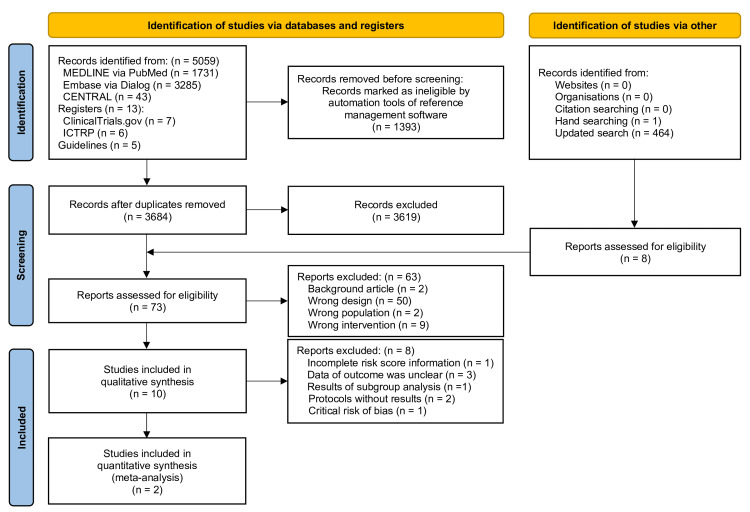
PRISMA 2020 flow diagram of the literature search results CENTRAL: Cochrane Central Register of Controlled Trials; ICTRP: International Clinical Trials Registry Platform.

Figure 1 shows a PRISMA flowchart of the study selection process. Of the 4,149 articles identified after duplicates were removed, 73 were selected for full-text screening. After the full-text screening, 63 reports were excluded (Appendix Table 4); overall, ten studies were included in the qualitative synthesis [10-16,31-33]. We excluded eight reports: one with incomplete risk score information [12], three with unclear outcome data [13,14,33], one with subgroup analysis result [15], one with critical risk of bias [16] (Appendix Table 5), and two unpublished trials [31,32]. Therefore, two RCTs that fulfilled all the eligibility criteria in the meta-analysis were included [10,11].

Characteristics of the Studies

Table 1 shows the characteristics of the ten studies included in the qualitative synthesis (2192 participants). Of the ten studies, we included two RCTs, six retrospective cohort studies, and two protocols [10-16,31-33]. Regarding the type of surgery-first approach assessed, eight were studies on second uterine curettage, whereas two were studies on hysterectomy. The following four chemotherapy regimens were used in the studies: MTX, MTX with folic acid support, actinomycin D, and single-agent chemotherapy. We extracted data on the main outcomes of the studies. Data on remission, the mean number of chemotherapy courses required to cure, and adverse events were extracted from three, four, and one study, respectively. In total, 138 patients were included in the two studies (69 who underwent a second uterine curettage and 69 who were treated using a chemotherapy-first approach) [10,11].

**Table 1:** Characteristics of the studies included in the qualitative synthesis ^a^Not reported; ^b^mixed chemotherapy includes various regimens, including administration of MTX, MTX-FA, and actinomycin D. CI: confidence interval; MD: mean difference; RR: risk ratio; RCT: randomized controlled trial; MTX: methotrexate; FA: folic acid; N/A: not applicable.

Author (year)	Design	Region	Intervention		Total number of patients	Reported main outcomes					
						Remission		The mean number of chemotherapy courses required to cure		Adverse events	
			Arm 1 (surgery-first approach)	Arm 2 (Chemotherapy-first approach)		RR	95% CI	MD	95% CI	RR	95% CI
Ayatollahi et al. [10]	RCT	Iran	Second uterine curettage + MTX every week	MTX every week	52	N/A		−5.16	−6.67, −3.65	N/A	
Hemida et al. [11]	RCT	Egypt	Second uterine curettage + MTX-FA every fortnight	MTX-FA every fortnight	86	1.00	0.96, 1.05	−0.60	−1.55, 0.35	0.87	0.47, 1.60
Ahmadzadeh et al. [16]	Retrospective cohort study	Iran	Second uterine curettage + single-agent chemotherapy^a^	Single-agent chemotherapy^a^	148	N/A		−3.59	−4.21, −2,97	N/A	
van Trommel et al. [12]	Retrospective cohort study	Netherlands	Second uterine curettage + MTX-FA every fortnight	MTX-FA every fortnight	294	1.00	0.98, 1.02	−1.00	−1.64, −0.36	N/A	
Growdon et al. [13]	Retrospective cohort study	USA	Second uterine curettage +mixed chemotherapy ^b ^	Mixed chemotherapy^b^	150	N/A					
Aminimoghaddam et al. [33]	Retrospective cohort study	Iran	Second uterine curettage + MTX every fortnight	MTX every fortnight	50	N/A					
Bolze et al. [14]	Retrospective cohort study	France	Hysterectomy +MTX	MTX	992	N/A					
Ramesan et al. [15]	Retrospective cohort study	India	Hysterectomy +mixed chemotherapy^b^	Mixed chemotherapy^b^	46	1.00	0.60, 1.66	N/A			
Weiguo et al. [31]	RCT (protocol)	China	Second uterine curettage + MTX every fortnight	MTX every fortnight	224	N/A					
Marcio et al. [32]	RCT (protocol)	International	Second uterine curettage + MTX-FA every fortnight	MTX-FA every fortnight	150	N/A					

Primary Outcomes

**Figure 2:**
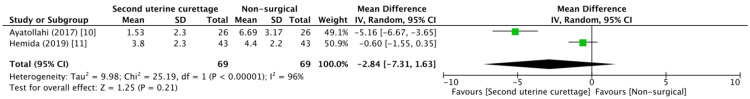
Forest plot of the comparison of the mean numbers of chemotherapy courses required to cure

**Figure 3:**
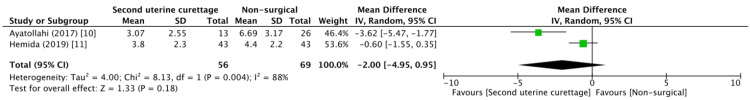
Forest plot of the comparison of the mean number of chemotherapy courses required to cure; subgroup analysis was performed by the exclusion of studies on second uterine curettage without chemotherapy

**Figure 4:**
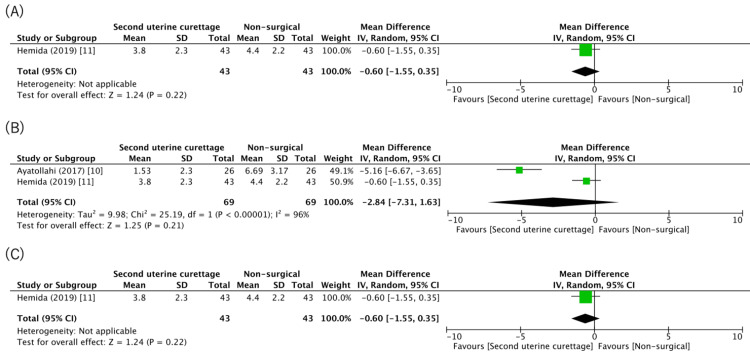
Sensitivity analysis of the mean number of chemotherapy courses required to cure (A) Sensitivity analysis of the mean number of chemotherapy courses required for treatment after the exclusion of studies in which imputed statistics were used; (B) sensitivity analysis of the mean number of chemotherapy courses required for treatment after exclusion of studies conducted using current non-standard chemotherapy (except for methotrexate and actinomycin D); (C) sensitivity analysis of the mean number of chemotherapy courses required for treatment after the exclusion of studies with a high risk of bias.

The summary of findings (Table 2) indicates that, compared to the chemotherapy-first approach, second uterine curettage may result in little to no difference in remission (one study, 84 patients: RR 1.00, 95% CI 0.96 to 1.05; low certainty) [11]. The evidence is very uncertain about the effect of a second uterine curettage on the mean number of chemotherapy courses required to cure (two studies, 138 patients: MD −2.84, 95% CI −7.31, 1.63; I2 = 96%; very low certainty) (Figure 2) [10,11]. Since the authors of the study by Ayatollahi et al. did not reply when contacted, we imputed the SD for the study based on another study [23]. The baseline indications for adjuvant chemotherapy, FIGO/WHO staging and risk score, different drug regimens, and pre-intervention hCG level showed considerable clinical heterogeneity. However, we did not downgrade the level of inconsistency since the effects of the two studies were similar (in favor of second uterine curettage). We conducted a subgroup analysis to exclude second-uterine curettage without chemotherapy (Figure 3). The sensitivity analyses are shown in Figure 4A-4C. Second uterine curettage may result in a slight reduction in adverse events (one study, 86 patients: RR 0.87, 95% CI 0.47 to 1.60; low certainty) [11], and the only adverse event reported for a second uterine curettage was drug toxicity, wherein one emergency curettage and one emergency hysterectomy were associated with the chemotherapy-first approach.

**Table 2:** Summary of findings ^a^The risk in the intervention group (and its 95% confidence interval) was based on the assumed risk in the comparison group and the relative effect of the intervention (and its 95% CI). ^b^Grading of recommendations assessment, development and evaluation working group grades of evidence. High certainty: We are confident that the true effect lies close to the estimated effect. Moderate certainty: We are moderately confident in the effect estimate (the true effect is likely to be close to the estimate; however, there is the possibility that it is substantially different). Low certainty: Our confidence in the effect estimate was limited (the true effect may differ substantially from the effect estimate). Very low certainty: We have little confidence in the effect estimate (the true effect is likely to differ substantially from the effect estimate). ^c^Downgraded by two levels for very serious imprecision (failure to meet optimal information size). ^d^Downgraded by one level for serious limitations in the study design (one study had a high risk of deviation from intended interventions and missing outcome data) and by two levels for very serious imprecision (95% CI is wide). Chemotherapy-first approaches included emergency curettage (n=1) and hysterectomy (n=1). CI: confidence interval; MD: mean difference; RR: risk ratio; RCT: randomized controlled trial.

Outcomes	Anticipated absolute effects ^a^ (95% CI)		Relative effect (95% CI)	Number of participants (studies)	Certainty of the evidence (GRADE)^b^	Comments
	Risk with chemotherapy-first approach	Risk with second uterine curettage				
Remission	1,000 per 1,000	1000 per 1,000 (960 to 1,000)	RR 1.00 (0.96 to 1.05)	84 (1 RCT)	⨁⨁◯◯ Low ^c^	Second uterine curettage may result in little to no difference in remission.
Mean number of chemotherapy courses required to cure		MD 2.84 lower (-7.31 lower to 1.63 higher)	-	138 (2 RCTs)	⨁◯◯◯ Very low ^d^	The evidence is very uncertain about the effect of second uterine curettage on mean number of chemotherapy courses required to cure.
Adverse events	349 per 1,000	303 per 1,000 (164 to 558)	RR 0.87 (0.47 to 1.60)	86 (1 RCT)	⨁⨁◯◯ Low ^c^	Second uterine curettage may result in a slight reduction in adverse events.

Quality Assessment

**Figure 5:**
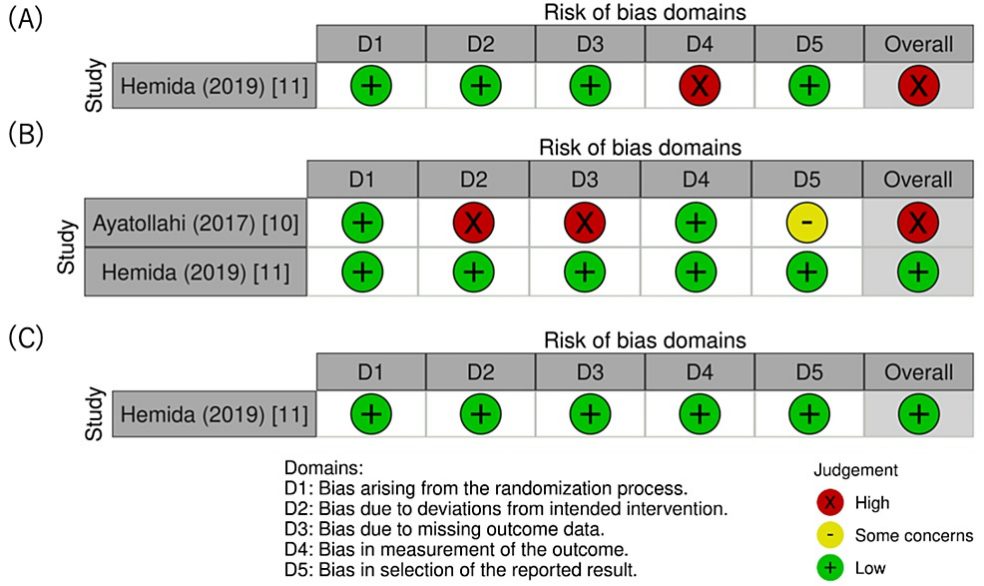
Risk-of-bias graph for primary outcomes (A) Risk-of-bias graph for studies on second uterine curettage in which remission was evaluated; (B) risk-of-bias graph for studies on second uterine curettage in which the mean number of chemotherapy courses required for treatment was evaluated; (C) risk-of-bias graph for studies on second uterine curettage in which adverse events were evaluated.

**Figure 6:**
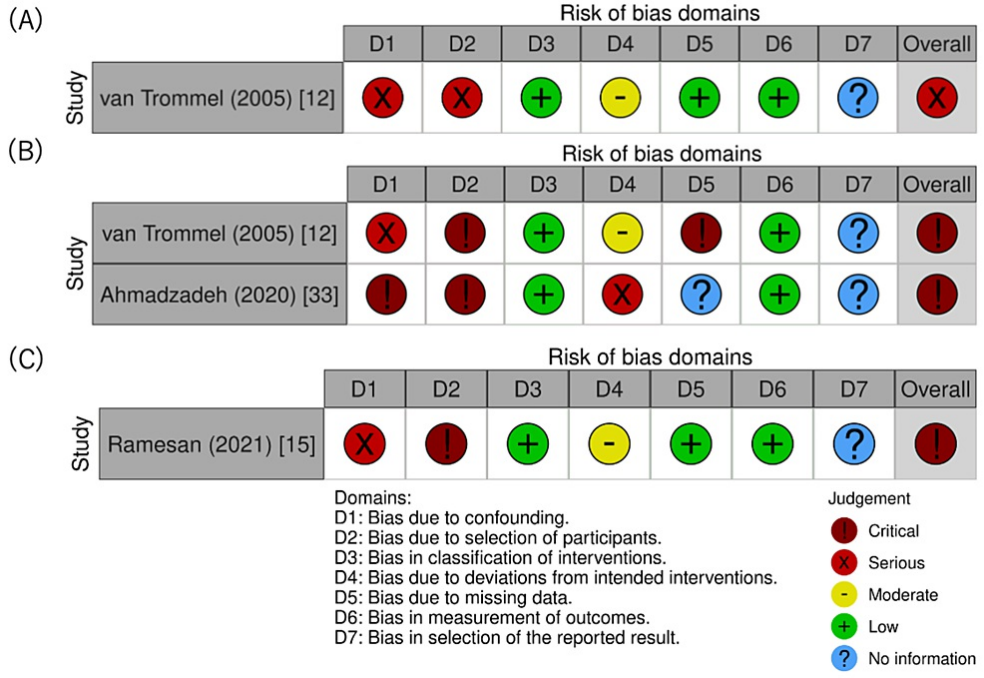
Risk of bias in non-randomized studies of interventions (A) ROBINS-I graph for a study on second uterine curettage in which remission was evaluated; (B) ROBINS-I graph for a study on second uterine curettage in which the mean number of chemotherapy courses required for treatment was evaluated; (C) ROBINS-I graph for a study on a hysterectomy in which remission was evaluated.

The results of assessing the risks of bias in the RCTs are outlined in Figure 5A-5C. Regarding the remission outcome, the risk of bias in the included RCTs was low. Among the studies included in the meta-analysis, one RCT was well-designed and had a low risk of bias [11]. However, the other RCT [10] was unclear about analytical methods, such as blinding of assessors from the main text, and the protocol could not be obtained, resulting in a high risk of bias in domain 2. Additionally, owing to the high proportion of missing data, domain 3 also had a high risk of bias, leading us to evaluate the overall risk of bias as high. For the outcomes of adverse events, the assessors were not blinded; therefore, there was a possibility that the assessment of outcomes could be affected [11]. The results of assessing the risks of bias in the retrospective cohort studies are shown in Figure 6A-6C.

Discussion

Summary of Main Results

Our findings suggest that, compared with the chemotherapy-first approach, second uterine curettage plus chemotherapy, as required for treating low-risk GTN, may not increase remission. Although second uterine curettage may slightly reduce adverse events, its effect on the mean number of chemotherapy courses required to cure is unclear. We discovered that, compared with the chemotherapy-first approach, the clinical outcomes of hysterectomy plus chemotherapy were unclear owing to insufficient data.

Overall Completeness and Applicability of Evidence

This study is the first systematic review and meta-analysis of RCTs and comparative observational studies on this topic. It provides insights into the surgery-first approach for low-risk GTN. While the results are applicable to patients with low-risk GTN considering post-remission pregnancy or fertility preservation, the certainty of the evidence is low or very low owing to a lack of data and potential biases. In addition, there are no RCTs or non-comparative observational studies on the effect of hysterectomy on low-risk GTN to date. However, since the surgery-first approach for treating low-risk GTN could be performed according to individual patient preferences and conditions, doctors should present additional options to meet the needs of patients with low-risk GTN, including pregnancy following remission or fertility preservation.

Quality of the Evidence

The findings of this study suggest that second uterine curettage is comparable to chemotherapy as a first-line treatment option for low-risk GTN; however, the certainty of this evidence is low or very low. In addition, there were no significant differences between the second uterine curettage approach and the chemotherapy-first approach in terms of the primary outcomes. The outcomes of hysterectomy plus chemotherapy were unclear due to insufficient data.

Potential Biases in the Review Process

This study has some limitations that may have introduced bias into the review process. First, we did not include studies in which chemotherapy regimens were used as interventions in our search strategy, thus potentially overlooking eligible studies. Second, we could not obtain a protocol for one of the included RCTs [10]. This resulted in a lack of clarity concerning whether the number of chemotherapy courses reported included treatment after second-line therapy or whether all patients achieved remission after first-line chemotherapy. Such ambiguities may have led to heterogeneity in our study results. Finally, there was variability in patient background, particularly in the average hCG levels (3,000 mIU/mL vs. 10,000-13,000 mIU/mL) between the two RCTs [10,11]. Moreover, the inclusion of cases with incomplete primary surgery for hydatidiform moles based solely on the FIGO hCG criteria may have biased our results. The diagnostic criteria and practices for low-risk GTN vary across hospitals, which may have increased the heterogeneity of our study findings.

Agreements and Disagreements with Other Studies or Reviews

We could not clarify the efficacy of a second uterine curettage compared with the chemotherapy-first approach regarding beneficial outcomes. Our results indicate that, compared with the chemotherapy-first approach, second uterine curettage results in little to no difference in remission; this is because almost all patients with low-risk GTN achieve complete remission with chemotherapy, multi-agent regimens, or surgical interventions [4,5].

Furthermore, the effect of second uterine curettage on the mean number of chemotherapy courses required to cure could not be clarified because the certainty of the evidence was very low. In one of the included RCTs [11], all the patients were treated using adjuvant chemotherapy, which commenced within 24 hours of the second uterine curettage. Several non-comparative studies have demonstrated that 40-68% of patients achieve remission with only a second uterine curettage as a first-line treatment [16,32,34]. This was consistent with the 50% remission achieved in the other included RCT [10]. Therefore, a second uterine curettage may reduce the mean number of chemotherapy courses required to cure GTN, as its outcome could determine whether adjuvant chemotherapy is required after postoperative hCG monitoring [12]. Additionally, since patients who achieved remission had to use contraception for at least 12 months of surveillance for relapse [1,6,7], second uterine curettage may be beneficial for patients who wish to become pregnant soon after remission. Among patients with GTN who received standard chemotherapeutic regimens, the pregnancy rate was reported to be 97% for women who wished to become pregnant [35]. Further research may reveal second uterine curettage as the treatment of choice, as it may reduce the need for chemotherapy courses and provide more options to cater to a patient’s fertility needs.

Compared with the chemotherapy-first approach, the second uterine curettage may reduce adverse events, including uterine perforation. The drug toxicity would not occur if only second-uterine curettage were sufficient for remission [34]. In one RCT included in our meta-analysis that reported drug toxicity, grade 2 adverse events were less frequent in the second uterine curettage plus chemotherapy group [11]. However, a previous study reported that 1.6% of patients required blood transfusions. As such, caution regarding potential blood loss is necessary when performing a second uterine curettage, given that GTN is a vascular-rich tumor [34]. Nevertheless, if a second uterine curettage can be performed safely, it could offer potential benefits for patients with persistent vaginal bleeding because removing residual trophoblastic tissue through the second uterine curettage will reduce persistent uterine bleeding, thereby avoiding emergency surgical interventions and admissions [12,34,36,37]. Thus, a second uterine curettage could be an additional option according to individual patient preference and conditions, including the presence of residual uterine tissue, heavy vaginal bleeding, and the desire to avoid chemotherapy. In terms of complications of uterine curettage, no uterine perforations were observed owing to the small sample sizes of the included RCTs [10,11]. The reported incidence of uterine perforation during the second uterine curettage ranges from 1.7% to 8.0% [12,34,38]. However, intraoperative ultrasonography during uterine curettage reduces the incidence of uterine perforations [39], and there has been a trend toward using suction curettage instead of sharp curettage, which may also contribute to a decrease in uterine perforations [36]. Therefore, a second uterine curettage for the treatment of low-risk GTN may be safe and reduce adverse events, especially when coupled with intraoperative ultrasonography and suction curettage.

Although this study could not clarify the effect of hysterectomy compared with chemotherapy as a first-line treatment for low-risk GTN, several guidelines recommend that hysterectomy may be an alternative primary treatment for patients with low-risk GTN who do not wish to preserve fertility [6,9]. In some non-comparative studies, patients with low-risk GTN safely underwent adjuvant chemotherapy without perioperative complications [37] and achieved remission with only hysterectomy as the first-line treatment [14,40,41]. Moreover, a hysterectomy may reduce the number of chemotherapy courses required to cure low-risk non-metastatic GTN [41]. Therefore, physicians should present hysterectomy as a treatment option after evaluating the patient’s desire for fertility preservation at the start of treatment [1,6].

## Conclusions

In conclusion, while our findings suggest surgery-first approaches as potential treatment options for low-risk GTN, further high-quality, well-conducted studies are required to substantiate this finding. Such research will help establish more definitive evidence, improve guidance for patient care in the context of fertility needs, and may provide suitable options for patients with low-risk GTN.

## Appendices

**Table 3:** Search strategy

Search database	Search strategy
MEDLINE	#1 Trophoblastic Neoplasms[mh]
	#2 gestational trophoblastic[tiab]
	#3 postmolar [tiab]
	#4 trophoblastic cancer*[tiab]
	#5 trophoblastic neoplas*[tiab]
	#6 trophoblastic tumor*[tiab]
	#7 trophoblastic tumour*[tiab]
	#8 trophoblastic disease*[tiab]
	#9 choriocarcinoma*[tiab]
	#10 hydatidiform mole* [tiab]
	#11 invasive mole*[tiab]
	#12 molar[tiab]
	#13 pregnanc*[tiab]
	#14 #12 AND #13
	#15 #1 OR #2 OR #3 OR #4 OR #5 OR #6 OR #7 OR #8 OR #9 OR #10 OR #11 OR #14
	#16 Gynecologic Surgical Procedures[mh]
	#17 Curettage[mh]
	#18 hysterectomy[tiab]
	#19 curettage[tiab]
	#20 evacuat*[tiab]
	#21 #16 OR #17 OR #18 OR #19 OR #20
	#22 #15 AND #21
EMBASE (Dialog)	S1 “EMB.EXACT.EXPLODE(“trophoblastic tumor”)
	S2 ab(gestational trophoblastic) OR ti(gestational trophoblastic)
	S3 ab(postmolar) OR ti(postmolar)
	S4 ab(trophoblastic cancer*) OR ti(trophoblastic cancer*)
	S5 ab(trophoblastic neoplas*) OR ti(trophoblastic neoplas*)
	S6 ab(trophoblastic tumor*) OR ti(trophoblastic tumor*)
	S7 ab(trophoblastic tumour*) OR ti(trophoblastic tumour*)
	S8 ab(trophoblastic disease*) OR ti(trophoblastic disease*)
	S9 ab(choriocarcinoma*) OR ti(choriocarcinoma*)
	S10 ab(hydatidiform mole*) OR ti(hydatidiform mole*)
	S11 ab(invasive mole*) OR ti(invasive mole*)
	S12 ab(molar) OR ti(molar)
	S13 ab(pregnanc*) OR ti(pregnanc*)
	S14 S12 AND S13
	S15 S1 OR S2 OR S3 OR S4 OR S5 OR S6 OR S7 OR S8 OR S9 OR S10 OR S11 OR S14
	S16 “EMB.EXACT.EXPLODE(“hysterectomy”)
	S17 “EMB.EXACT.EXPLODE(“Curettage”)
	S18 ab(hysterectomy) OR ti(hysterectomy)
	S19 ab(curettage) OR ti(curettage)
	S20 ab(evacuat*) OR ti(evacuat*)
	S21 S16 OR S17 OR S18 OR S19 OR S20
	S22 S15 AND S21
Cochrane Central Register of Controlled Trials (Cochrane Library)	#1 MeSH descriptor: [Trophoblastic Neoplasms] explode all trees
	#2 (“gestational trophoblastic”):ti,ab,kw
	#3 (postmolar):ti,ab,kw
	#4 ((trophoblastic near/5 (cancer* or neoplas* or tumor* or tumour* or disease*))):ti,ab,kw
	#5 (choriocarcinoma*):ti,ab,kw
	#6 (((hydatid* or invasive) near/5 mole*)):ti,ab,kw
	#7 (molar near/5 pregnanc*):ti,ab,kw
	#8 #1 OR #2 OR #3 OR #4 OR #5 OR #6 OR #7
	#9 MeSH descriptor: [Hysterectomy] explode all trees
	#10 (hysterectomy):ti,ab,kw
	#11 MeSH descriptor: [Curettage] explode all trees
	#12 (curettage):ti,ab,kw
	#13 (evacuat*):ti,ab,kw
	#14 #9 OR #10 OR #11 OR #12 OR #13
	#15 #8 AND #14
ClinicalTrials.gov	Condition or disease: Trophoblastic Neoplasms OR gestational trophoblastic OR postmolar
	Intervention: Hysterectomy OR Curettage OR evacuation
International Clinical Trials Registry Platform	Conditions: Trophoblastic Neoplasms OR gestational trophoblastic OR postmolar
	Intervention: Hysterectomy OR Curettage OR evacuation

**Table 4:** List of studies excluded after full-text screening

Title	Journal	Reason for exclusion
Serum human chorionic gonadotropin regression pattern in persistent trophoblastic disease during chemotherapy	Journal of the Medical Association of Thailand Chotmaihet thangphaet	Wrong study design
Adjuvant hysterectomy in low-risk gestational trophoblastic disease	Obstetrics and Gynecology	Wrong intervention
Role of hysterectomy in management of gestational trophoblastic disease	Gynecologic Oncology	Wrong study design
Gestational trophoblastic diseases. Apropos of 105 cases.	Gynecologie, Obstetrique and Fertilite	Wrong study design
The role of repeat uterine evacuation in the management of persistent gestational trophoblastic disease	Gynecologic Oncology	Wrong study design
Gestational trophoblastic tumors after molar pregnancies	Revue du Praticien – Gynecologie et Obstetrique	Background article
The role of repeat uterine evacuation in trophoblast disease	Gynecologic Oncology	Wrong study design
The role of adjuvant surgery in the management of gestational trophoblastic neoplasia	The Journal of Reproductive Medicine	Wrong study design
Role of hysterectomy in managing persistent gestational trophoblastic disease	The Journal of Reproductive Medicine	Wrong study design
Hysterectomy in gestational trophoblastic neoplasia: Chiang Mai University Hospital’s experience	Asian Pacific Journal of Cancer Prevention	Wrong study design
The evolving role of hysterectomy in gestational trophoblastic neoplasia at the New England Trophoblastic Disease Center	The Journal of Reproductive Medicine	Wrong study design
Gestational trophoblastic disease: Regional perspective in Rio de Janeiro, Brazil	The Journal of Reproductive Medicine	Wrong study design
Major surgeries performed for gestational trophoblastic neoplasms in a teaching hospital in Tehran, Iran	Journal of Gynecologic Oncology	Wrong intervention
The impact of uterine re-curettage, pre-evacuation and week-one level of hCG on the number of chemotherapy courses in treatment of post molar GTN	Journal of Experimental Therapeutics & Oncology	Wrong population
The impact of uterine re-curettage on the number of chemotherapy courses in treatment of post molar GTN	International Journal of Gynecological Cancer	Background article
Role of minimal access surgery in gestational trophoblastic disease	World Journal of Laparoscopic Surgery	Wrong study design
Major surgeries performed in gestational trophoblastic neoplasms in a teaching hospital Tehran, Iran	International Journal of Gynecological Cancer	Wrong study design
The role of hysterectomy In the treatment of gestational trophoblastic neoplazias: A single center experience of 17 years	Turk Jinekoloji ve Obstetrik Dernegi Dergisi	Wrong study design
The impact of uterine re-curettage on the number of chemotherapy courses in treatment of post molar gestational trophoblastic neoplasia. A randomized controlled study	https://trialsearch.who.int/Trial2.aspx?TrialID=NTR3390	Wrong study design
Role of hysterectomy in the management of patients with gestational trophoblastic neoplasia: Importance of receiving treatment in reference centers	The Journal of Reproductive Medicine	Wrong study design
Clinical epidemiology and management of gestational trophoblastic neoplasia in Hungary in the past 34 years	The Journal of Reproductive Medicine	Wrong study design
Treatment outcome of metastatic gestational trophoblastic neoplasia in Chiang Mai University Hospital	International Journal of Gynecological Cancer	Wrong study design
Major surgery in gestational trophoblastic neoplasms in Tehran, Iran, 1995-2005	International Journal of Gynecological Cancer	Wrong study design
Hysterectomy in case of low-risk gestational trophoblastic neoplasia	Oncologie	Wrong study design
Fertility-sparing partial hysterectomy for gestational trophoblastic neoplasia: an analysis of 36 cases	The Journal of Reproductive Medicine	Wrong intervention
Evaluation of response rate to surgery of gestational trophoblastic neoplasia (GTN)	International Journal of Gynecological Cancer	Wrong study design
Clinical response to a second uterine curettage in patients with low-risk gestational trophoblastic disease: a pilot study	The Journal of Reproductive Medicine	Wrong study design
A phase II study to determine the response to second curettage as initial management for persistent low-risk non-metastatic gestational trophoblastic neoplasia: A gynecologic oncology group study	International Journal of Gynecological Cancer	Wrong study design
Single center analysis of gestational trophoblastic disease in Turkey	International Journal of Gynecological Cancer	Wrong study design
Role of surgical therapy in the management of gestational trophoblastic neoplasia	Obstetrics & Gynecology Science	Wrong intervention
Belgian register for gestational trophoblastic diseases: How does hysterectomy impact the management of gestational trophoblastic disease/gestational trophoblastic neoplasia. A retrospective analysis of 11 cases	International Journal of Gynecological Cancer	Wrong study design
The role of surgery in the management of gestational trophoblastic neoplasia The Hungarian experience	The Journal of Reproductive Medicine	Wrong study design
Second curettage for low-risk nonmetastatic gestational trophoblastic neoplasia	Obstetrics and Gynecology.	Wrong study design
Management and outcomes of patients with stage I and III low-risk gestational trophoblastic neoplasia treated in Sheffield, UK, from 1997-2006	Journal of Reproductive Medicine	Wrong study design
Evaluation of curative effect of re-curettage on the number of chemotherapy courses in low-risk persistent gestational trophoblastic disease; a pilot randomized clinical trial study	International Journal of Gynecological Cancer	Wrong study design
The Belgian registry for gestational trophoblastic diseases: Curative effect of a second curettage	International Journal of Gynecological Cancer	Wrong study design
The added value of hysterectomy in the management of gestational trophoblastic neoplasia	Gynecologic Oncology	Wrong population
Overview on gestational trophoblastic neoplasia (GTN) in Yangon General Hospital, Myanmar	Annals of Oncology	Wrong study design
Outcome of first-line hysterectomy for gestational trophoblastic neoplasia in patients no longer wishing to conceive and considered with isolated lung metastases: A series of 30 patients	International Journal of Gynecological Cancer	Wrong study design
Medical and surgical treatment for postmolar low-risk gestational trophoblastic neoplasia after failure of single-agent treatment	Journal of Reproductive Medicine	Wrong study design
Gestational trophoblastic neoplasia fertility outcomes and survival	Indian Journal of Gynecologic Oncology	Wrong intervention
Complete resection is essential in the surgical treatment of gestational trophoblastic neoplasia	International Journal of Gynecological Cancer	Wrong intervention
Clinical outcomes of gestational trophoblastic disease who underwent hysterectomy: A 12-year clinical experience at single institute	International Journal of Gynecological Cancer	Wrong study design
Second curettage for treatment of low-risk gestational trophoblastic neoplasia: A cost-effectiveness analysis	Gynecologic Oncology	Wrong study design
Role of surgery in the management of gestational trophoblastic neoplasia	Indian Journal of Gynecologic Oncology	Wrong study design
Reproductive outcomes after treatment of patients with gestational trophoblastic neoplasia	International Journal of Gynecological Cancer	Wrong study design
Persistent trophoblastic disease: Negative course of disease and prognostic factors	International Journal of Gynecological Cancer	Wrong study design
Outcome of different treatment modalities for gestational trophoblastic neoplasia in women at 40 years old or above: A multicenter retrospective study	International Journal of Gynecological Cancer	Wrong study design
Gestational trophoblastic neoplasia: Clinical presentation, treatment and outcomes	International Journal of Gynecological Cancer	Wrong study design
Application of hysterectomy in treatment of trophoblastic neoplasia	Annals of Oncology	Wrong intervention
The impact of previous cesarean section (C/S) on the risk for post-molar gestational trophoblastic neoplasia (GTN)	Gynecologic Oncology	Wrong study design
Second curettage for treatment of low-risk gestational trophoblastic neoplasia: A Cost-effectiveness analysis	Gynecologic Oncology	Wrong study design
Cost-effectiveness of second curettage for treatment of low-risk non-metastatic gestational trophoblastic neoplasia	Gynecologic Oncology	Wrong study design
Clinical, socioeconomic characteristics, treatment and reproductive outcomes of patients with gestational trophoblastic neoplasia at a tertiary care hospital in India	Gynecologic Oncology	Wrong study design
Outcomes of minimally invasive versus open abdominal hysterectomy in patients with gestational trophoblastic disease	Gynecologic Oncology	Wrong intervention
Does hysteroscopy in women with persistent gestational trophoblastic disease reduce the need for chemotherapy? A prospective, single-arm, clinical trial pilot study	Gynecological Surgery	Wrong study design
Curative effect of second curettage for treatment of gestational trophoblastic disease: Results of the Belgian registry for gestational trophoblastic disease	European Journal of Obstetrics, Gynecology, and Reproductive Biology	Wrong study design
Gestational trophoblastic disease: A fifteen – year experience of a single tertiary institution	International Journal of Gynecological Cancer	Wrong design
Management and outcome of gestational trophoblastic disease in a Tunisian public hospital	International Journal of Gynecological Cancer	Wrong design
Gestational trophoblastic neoplasia: A Tunisian retrospective study	International Journal of Gynecological Cancer	Wrong design
Fertility-sparing, surgical interventions for low-risk, non-metastatic gestational trophoblastic neoplasia	Cochrane Database of Systematic Reviews	Wrong design
Gestational trophoblastic disease in Portugal: retrospective analysis of the last 10 years in two institutions	International Journal of Gynecological Cancer	Wrong design
The diagnostics and treatment of low-risk gestational trophoblastic neoplasia (GTN): 42-year experience	European Journal of Gynecological Oncology	Wrong intervention

**Table 5:** Characteristics of the studies excluded from the quantitative synthesis GTN: gestational trophoblastic neoplasia; FIGO: International Federation of Gynecology and Obstetrics; WHO: World Health Organization.

Study	Reason for exclusion	Comments
van Trommel et al. [12]	Incomplete risk score information	Low-risk GTN was defined using a different risk scoring system, which was similar to the FIGO/WHO staging and risk scoring system. Moreover, data on five items of the FIGO/WHO staging system and the risk scores for more than 30% of cases were missing.
Growdon et al. [13]	Unclear outcomes data	We could extract information on the number of patients with low-risk GTN who underwent second uterine curettage; however, we could not retrieve information on the primary outcome of the study because the authors did not reply when contacted.
Ahmadzadeh et al. [16]	Critical risk of bias	Although inclusion criteria were met, we excluded this study in the quantitative synthesis (meta-analysis) because the critical risk of bias was assessed using Risk Of Bias In Non-randomized Studies of Interventions (ROBINS-I).
Bolze et al. [14]	Unclear outcome data	We could extract data on the number of patients with low-risk GTN who underwent hysterectomy; however, we could not extract details of the primary outcome of the study because the authors did not reply when contacted.
Ramesan et al. [15]	Results of subgroup analysis	The outcomes of hysterectomy in low-risk and high-risk GTN patients were compared in this study. However, we could only extract data on the number of hysterectomies plus chemotherapy performed as required and the number of chemotherapy sessions received by low-risk GTN patients.
Aminimoghaddam et al. [33]	Unclear outcomes data	We could extract information on the number of patients with low-risk GTN who underwent second uterine curettage; however, we could not retrieve information on the primary outcome of the study because the authors did not reply when contacted.
